# An integrated analysis of the SOX2 microRNA response program in human pluripotent and nullipotent stem cell lines

**DOI:** 10.1186/1471-2164-15-711

**Published:** 2014-08-25

**Authors:** Sebastian F Vencken, Praveen Sethupathy, Gordon Blackshields, Cathy Spillane, Salah Elbaruni, Orla Sheils, Michael F Gallagher, John J O’Leary

**Affiliations:** Department of Histopathology, Trinity College Dublin, Sir Patrick Dun Research Laboratory, St. James’s Hospital, Dublin, Ireland; The Coombe Women and Infants University Hospital, Dublin, Ireland; Department of Genetics, University of North Carolina at Chapel Hill, Chapel Hill, North Carolina USA

**Keywords:** SOX2, microRNA, Embryonic stem cell, Embryonal carcinoma, Pluripotency, EMT

## Abstract

**Background:**

SOX2 is a core component of the transcriptional network responsible for maintaining embryonal carcinoma cells (ECCs) in a pluripotent, undifferentiated state of self-renewal. As such, SOX2 is an oncogenic transcription factor and crucial cancer stem cell (CSC) biomarker in embryonal carcinoma and, as more recently found, in the stem-like cancer cell component of many other malignancies. SOX2 is furthermore a crucial factor in the maintenance of adult stem cell phenotypes and has additional roles in cell fate determination. The SOX2-linked microRNA (miRNA) transcriptome and regulome has not yet been fully defined in human pluripotent cells or CSCs. To improve our understanding of the SOX2-linked miRNA regulatory network as a contribution to the phenotype of these cell types, we used high-throughput differential miRNA and gene expression analysis combined with existing genome-wide SOX2 chromatin immunoprecipitation (ChIP) data to map the SOX2 miRNA transcriptome in two human embryonal carcinoma cell (hECC) lines.

**Results:**

Whole-microRNAome and genome analysis of SOX2-silenced hECCs revealed many miRNAs regulated by SOX2, including several with highly characterised functions in both cancer and embryonic stem cell (ESC) biology. We subsequently performed genome-wide differential expression analysis and applied a Monte Carlo simulation algorithm and target prediction to identify a SOX2-linked miRNA regulome, which was strongly enriched with epithelial-to-mesenchymal transition (EMT) markers. Additionally, several deregulated miRNAs important to EMT processes had SOX2 binding sites in their promoter regions.

**Conclusion:**

In ESC-like CSCs, SOX2 regulates a large miRNA network that regulates and interlinks the expression of crucial genes involved in EMT.

**Electronic supplementary material:**

The online version of this article (doi:10.1186/1471-2164-15-711) contains supplementary material, which is available to authorized users.

## Background

SOX2 is a member of the SRY-related HMG-box (SOX) transcription factor family with a set of well-established and diverse roles in stem cell potency and maintenance, embryonic development and cancer [1–10]. It regulates extensive and often divergent transcriptional networks across different cell types [1, 2, 11, 12]. SOX2 is best known as a core pluripotency factor, maintaining the undifferentiated phenotype of pluripotent stem cells, and is closely co-regulated alongside core pluripotency factors OCT4 and NANOG in undifferentiated embryonic stem cells (ESCs), embryonal carcinoma cells (ECCs) and induced pluripotent stem cells (iPSCs) [8, 13, 14]. Loss of SOX2 expression in these cell lines triggers their differentiation. More recently, SOX2 has been identified as a crucial player in the maintenance and differentiation of adult stem cells such as in neural stem cells [15]. As an oncogene, SOX2 has been implicated in many different malignancies of the central nervous, gastrointestinal, circulatory, respiratory, endocrine and skeletal systems, and also those of the skin, liver, gonads and breast [1–7, 10]. However, despite its oncogenic potential in numerous tumour types, the suppression of SOX2 has been reported as a hallmark of gastric carcinoma [16–18]. In malignancy growing evidence reveals SOX2 to be a central regulator of a tumourigenic, stem cell-like subpopulation of tumour cells, frequently referred to as cancer stem cells (CSCs), which are found to be responsible for the proliferative and invasive capacities of most tumour types [7]. Many of the genes regulated by SOX2 in normal stem cells are aberrantly regulated by this transcription factor in cancerous cells with a similar, albeit malignant phenotype.

MicroRNAs (miRNA) are a functional family of short (21–23 nt), non-protein coding RNA transcripts that primarily, but not exclusively, confer regulation of gene expression by targeting mRNAs for degradation or transient translational repression by post-transcriptionally binding these in a directed manner [19]. They are involved in the regulation of almost all cell processes and maintain cell homeostasis in both healthy and disease conditions. In cancer and CSCs, many miRNAs have been identified as tumour suppressive or oncogenic miRNAs (oncomiRs) [20]. Many of these miRNAs also have important regulatory functions in pluripotent cells, such as ESCs and ECCs, and in embryonic development [20, 21].

Although the SOX2 transcriptome of protein coding genes has been previously mapped in various cell types and tissues, including ECCs, with techniques such as chromatin immunoprecipitation (ChIP) and gene array profiling, no extensive SOX2-transcriptome analysis has been performed for miRNAs in human pluripotent cells [1, 2, 11, 12, 22–25]. Some insight into the SOX2-linked miRNAome in murine pluripotent cells has previously been provided by Marson *et al.* who performed an extensive ChIP-sequencing (ChIP-seq) analysis of SOX2-bound miRNA promoters in mouse ESCs [26]. Additionally, in a study of the SOX2 regulatory network in human ESCs (hESCs), Boyer *et al.* produced a limited set of exclusively intragenic miRNAs that were potentially regulated by the SOX2-binding sites within the promoter regions of their respective host genes [11]. However, both Marson *et al*. and Boyer *et al.* provide no SOX2 knock-down and miRNA expression analysis to functionally link this transcription factor to specific miRNAs. Fang *et al*. profiled genes and miRNAs regulated by SOX2 in glioblastoma multiforme (GBM) cells [1]. Notably absent from this study were an in-depth analysis of miRNAs directly regulated by SOX2 and a large scale combinatorial study of the gene target regulome of deregulated miRNAs in these cells.

To map the functions of this miRNA network in pluripotent cell and CSC biology, we silenced SOX2 in two human ECC (hECC) lines followed by a high-throughput expression analysis of expression changes in its associated miRNA network. To reveal genes potentially regulated by SOX2 through its linked miRNA regulome, we profiled whole-genome differential mRNA expression and applied a Monte Carlo algorithm to identify a subset of SOX2-regulated miRNAs that confer a significant regulatory signature on the differential gene expression profile in hECCs. This type of analysis has been performed before and is enabled by the finding that the majority of total miRNA-mediated gene suppression functions through mRNA degradation, thus functional miRNA activity can be determined by measuring the expression of their targets at the transcript level [27–36]. Finally, to identify candidate miRNAs with a direct transcriptional association to SOX2, we applied existing SOX2 ChIP-seq and miRNA promoter data to our differential expression profiles.

To study SOX2 in both a CSC and pluripotent cell context, we chose the 2102Ep and NTera-2 hECC lines, the CSC component of teratocarcinoma, a type of germ cell tumour. The NTera-2 cell line is pluripotent and is frequently used as cell model to study ectodermal differentiation [37]. 2102Ep cells are considered ‘nullipotent’ as they are resistant to retinoic acid-induced differentiation and form homogenous EC tumours when xenografted into mice [38, 39]. 2102Ep cell differentiation can however be induced by low-density growth or by silencing core pluripotency factors SOX2 and OCT4 [23, 38]. Because 2102Ep and NTera-2 cell lines are phenotypically similar to hESCs in an undifferentiated state and during early differentiation stages, they have previously been used as an alternative hESC model [14, 23, 38–43]. For this study, 2102Ep and NTera-2 cells could provide significant insights into the post-transcriptional regulatory functions of SOX2 towards both cancer stemness and embryonic stemness.

Based on parameters set in our analysis we present several novel miRNAs that are direct transcriptional targets of SOX2. Indirect targets of this transcription factor included, we found many pro and anti-malignant miRNAs that also have important functions in embryonic development. Statistical analysis of whole-genome differential mRNA expression revealed a distinct Type 1 epithelial-to-mesenchymal (EMT) signature in an miRNA/gene target regulatory network linked to SOX2.

## Results

### SOX2 knock-down in 2102Ep and NTera-2 cells results in many altered miRNAs involved in both cancer and embryonic development

**Figure 1:**
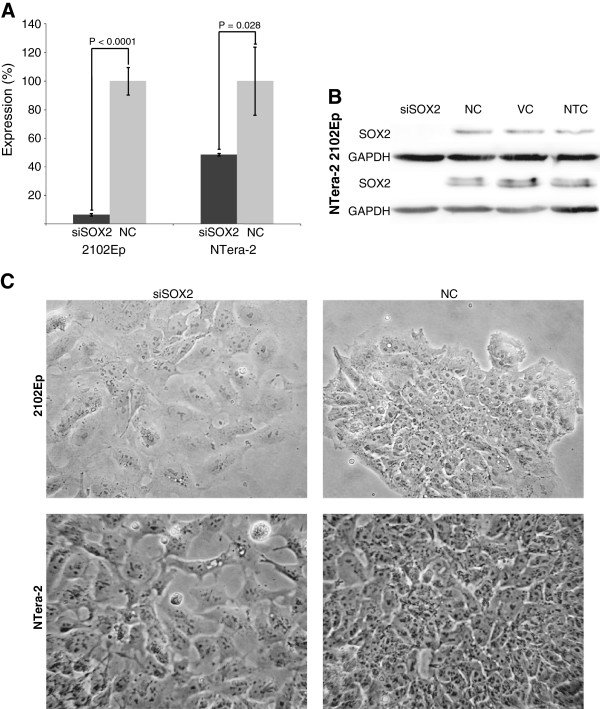
**Knock-down of SOX2 in 2102Ep and NTera-2 cells.** The induced RNAi of SOX2 mRNA by SOX2 siRNA (siSOX2) facilitated a substantial and significant down-regulation of this gene’s expression in the EC cell lines. **(A)** As determined by qRT-PCR, very little SOX2 mRNA expression remains (6.5%) in siSOX2-transfected 2102Ep cells, while the remaining expression in siSOX2-transfected NTera-2 cells is higher (48.5%). SOX2 expression was compared to that in cells transfected with a scrambled, non-targeting siRNA (NC), which was set at 100%. SOX2 expression was normalised to GAPDH expression. All experiments were performed in biological triplicate. **(B)** As determined by Western blot, no SOX2 protein could be detected in the siSOX2-transfected cells of either cell line when compared to the non-targeting siRNA-transfected controls (NC), vehicle controls (VC) or non-transfected controls (NTC). GAPDH protein expression was measured to account for equal loading and transfer. **(C)** RNAi phenotypes of 2102Ep and NTera-2 cells, four days after SOX2 siRNA transfection. SOX2-silenced EC cells have a flattened, enlarged and more dispersed morphology, compared to SC-transfected cells, which are, similarly to untreated cells (not shown), small, defined and grown in dense colonies.

SOX2 was selected for RNAi-mediated silencing in the two hECC lines and an siRNA with specific complementarity to SOX2 mRNA (siSOX2). Successful knock-down was confirmed by comparing SOX2 mRNA and protein expression with cells transfected with a non-targeting control (Figure 1A and Figure 1B). SOX2 mRNA was highly downregulated in 2102Ep cells, while in NTera-2 cells 49% reduced mRNA expression was reported. However, subsequent protein analysis showed that the SOX2 protein expression was completely eliminated, confirming successful knock-down.As shown in Figure 1C, distinct changes in cell morphology were recorded in 2102Ep and NTera-2 cells three days after siSOX2 transfection compared to cells transfected with a non-targeting siRNA control. For both cell lines, the cells had a flatter and larger appearance. They were also more evenly dispersed in monolayers, which differed from their normal, undifferentiated phenotype as densely packed colonies. This phenotypic change is characteristic of ECC differentiation.

**Figure 2:**
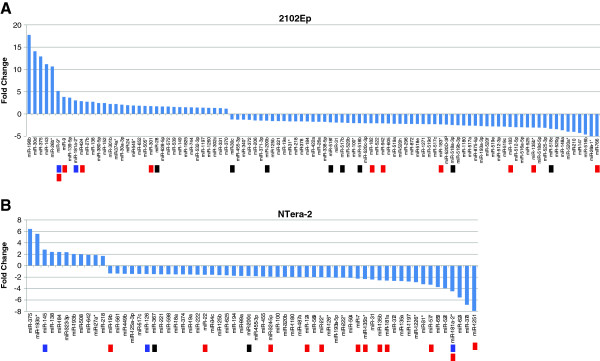
**Differential expression of miRNA in 2102Ep and NTera-2 cell lines after induced SOX2 silencing.** Total number of all significantly (P ≤ 0.05) deregulated miRNA three days after SOX2 knock-down in 2102Ep **(A)** and NTera-2 cells **(B)** normalised to the respective miRNA expression in cells transfected with a non-targeting control. 99 and 62 miRNAs were deregulated in 2102Ep and NTera-2 cells respectively. Both charts visualise a substantial bias towards miRNA downregulation after the knock-down of SOX2. These charts represent the mean expression values across three biological replicates. Blue markers indicate miRNAs that form an autoregulatory loop with SOX2. Black markers indicate miRNAs found to be significant master regulators of differential gene expression in hECCs. Red markers indicate miRNAs with SOX2 binding sites in their promoters as found in this study.

We profiled miRNA expression using high-throughput quantitative real-time PCR (qRT-PCR) arrays in 2102Ep and NTera-2 cells three days after transfecting them with siSOX2 or a non-targeting, scrambled siRNA control. This experiment tested the expression of 754 validated miRNAs, covering the majority of the Sanger miRBase v14 miRNA database [44]. From this set of miRNAs, 99 and 62 miRNAs were significantly (P ≤ 0.05) deregulated in 2102Ep and NTera-2 cells respectively (Figure 2 and Additional file 1: Table S1). These include the minor strand (star, ‘*’) miRNAs, which have increasingly been attributed with biological functions. The large majority of differentially expressed miRNA in both cell lines were downregulated, suggesting that SOX2 is predominantly a transcriptional activator of miRNAs in hECCs.

14 miRNAs were mutually deregulated (mir-1180, -125b, -135b*, -138, -19a, -221, -31, -31*, -372, -375, -378, -455-3p, -517c, -522), of which four were mutually deregulated by over 2-fold each (miR-135b*, -138, -375, -522). Another four miRNAs were oppositely regulated (miR-1197, -miR-181a-2*, -184, -218), with miR-181a-2* showing a large difference between 2102Ep (up) and NTera-2 cells (down). These results will be further detailed in following paragraphs.

Many deregulated miRNAs in 2102Ep and NTera-2 cells after SOX2 knock-down have previously reported oncogenic or tumour suppressive functions. In addition, many are actors in the maintenance of stem cell phenotypes or guide embryonic development and differentiation. We cross-analysed the deregulated miRNAs in both hECC lines with hESC miRNA data from Stadler *et al*. and the miRCancer database, a curated repository of oncomiRs and tumour suppressor miRNA [45, 46]. Table 1 contains a summary of deregulated miRNAs that have roles in cancer and also are enriched in hESCs or are differentially expressed during early hESC differentiation. This demonstrates great overlap of the deregulated miRNAs in our dataset and many miRNAs involved in cancer and stem cell biology.

**Table 1:** **Deregulated oncogenic or tumour suppressive miRNAs in 2102Ep and NTera-2 cells with an upregulated or downregulated profile in undifferentiated or differentiated hESC data from Stadler** ***et al.*** [45]

2102Ep	hESC undifferentiated		hESC differentiated	
**OncomiR**	*Downregulated*	miR-519c	*Upregulated*	*Downregualted*
	miR-19a	miR-519d	miR-24	miR-26a
	miR-512-5p	miR-520a*		miR-31
	miR-517a	miR-520b		miR-125b
	miR-517b	miR-520f		mir-182
	miR-517c	miR-520 g		miR-221
	miR-518b	miR-520 h		
	miR-518f			
	miR-525			
**Tumour Suppressor miRNA**	*Downregulated*		*Upregulated*	*Downregulated*
	miR-148a		miR-424	miR-26a
			miR-27b	miR-31
			miR-28	miR-125b
			miR-30d	
			miR-149	
			miR-331	
**NTera-2**	**hESC undifferentiated**		**hESC differentiated**	
**OncomiR**	*Downregulated*		*Upregulated*	*Downregulated*
	miR-18a		miR-193b	miR-22
	miR-19a			miR-125b
	miR-19b			miR-221
	miR-367			miR-222
	miR-374			
	miR-517c			
**Tumour Suppressor miRNA**			*Upregulated*	*Downregulated*
			miR-145	miR-22
			miR-193b	miR-31
				miR-125b
				miR-34c

The individual titles ‘Downregulated’ and ‘Upregulated’ indicate the differential expression of each miRNA after SOX2 knock-down in either 2102Ep or NTera-2 cell lines or both.

We also compared the miRNA profiles of the 2102Ep and NTera-2 cell lines in their native, undifferentiated state. This comparison produced 213 differentially regulated miRNA when both datasets are normalised to their respective global mean miRNA expression (Additional file 2: Table S2). This substantial difference in miRNA expression profiles could contribute to the divergence of differential miRNA expression profiles in 2102Ep and NTera-2 cells after SOX2 knock-down.

### Statistical identification of an enriched miRNA regulatory signature reveals the regulation of EMT-related genes

Current research has primarily focused on the direct action of SOX2 on its transcriptional target genes in the context of pluripotency, differentiation and cancer. To expand the picture of SOX2 regulation beyond this, we intended to identify SOX2-linked miRNAs that conferred a significant regulatory signature on the differential gene expression profile in hECCs after SOX2 knock-down.

For this approach, genome-wide gene expression arrays were used to generate differential gene expression profiles of the 2102Ep and NTera-2 cells from the same SOX2 knock-down samples used for miRNA profiling analysed against the same non-targeting control samples. All genes that were significantly ≥2-fold up or downregulated were included in further analysis and can be found in Additional file 3: Table S3. Using a Monte Carlo simulation algorithm combined with the miRNA target-prediction software, TargetScan 6.0, we analysed the gene expression data to identify deregulated miRNAs that target significantly more of the oppositely deregulated genes than would be expected by chance. This statistical method has previously been successfully applied to identify specific miRNAs as ‘master regulators’ of one-carbon metabolism-linked genes and lipid metabolism genes [47, 48].

**Figure 3:**
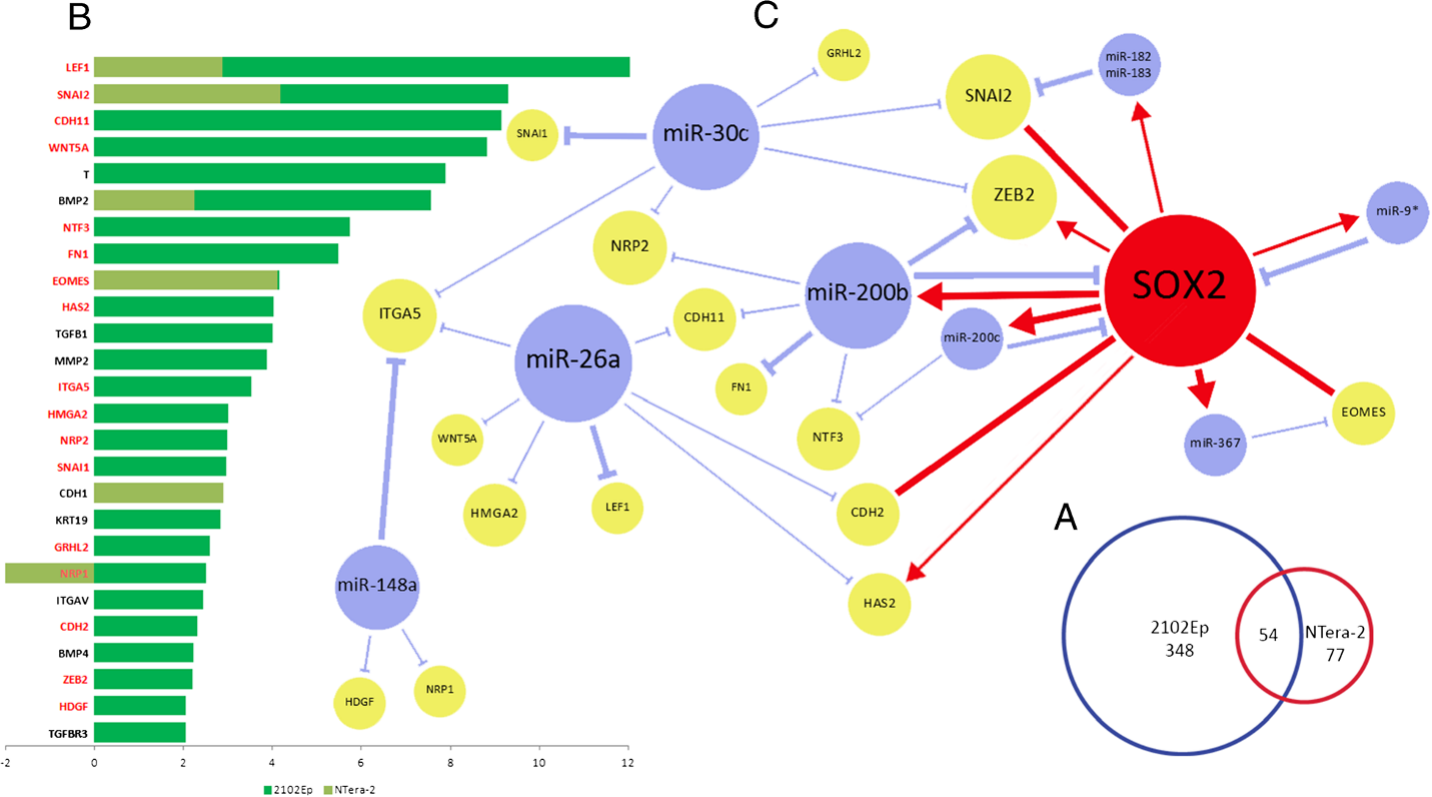
**EMT-specific miRNA-target relationships directly and indirectly regulated by SOX2 in 2102Ep and NTera-2 cells. (A)** This Venn diagram displays the exclusive and mutual expression of all ≥2-fold up or downregulated genes in 2102Ep and NTera-2 cells after SOX2 knock-down. Excluded from the mutual group were the four oppositely deregulated genes. As shown, despite a large overlap between the two cell lines, significantly more genes were deregulated in 2102Ep cells than NTera-2 cells. **(B)** This chart displays the expression levels of all 26 EMT genes differentially expressed in 2102Ep and NTera-2 cells combined. Those denoted in red are targets of the 11 miRNAs in Table 2 as predicted by our analysis. Several of these were previously validated. Together with SOX2 these miRNAs and targets form a complex network of EMT regulation **(C)**. The EMT network compiled from predicted miRNA and gene interaction data from this study and previously established interactions. The thick arrows represent previously validated functional relationships, which include those predicted by this study, while the thin arrows are novel relationships predicted by this study alone. Red arrows represent transcriptional control by SOX2. Stripes without arrowheads or bars represent the previously validated transcriptional activation by SOX2 of genes that could be transcriptionally inhibited by SOX2 in our study. SOX2 binding sites were also found in the promoter regions of HAS2 and ZEB2 as per the ChIP data from Lister *et al.* and Boyer *et al.*[11, 12]*.*

Whole-genome profiling revealed the deregulation of 402 genes in 2102Ep cells and 131 genes in NTera-2 cells, with 54 commonly deregulated genes between the two cell lines (Figure 3A and Additional file 3: Table S3). Gene ontology analysis revealed, particularly in 2102Ep cells, the deregulation of many markers of embryonic development, early tissue morphogenesis, cell differentiation and respective pathways involved, such as the Wnt pathway (Additional file 4: Table S4) [49]. An expected feature was the upregulation of 26 functional markers of mesodermal differentiation and EMT (see Figure 3B), with the exception of NRP1 in NTera-2 cells, which was downregulated in this cell line, but upregulated in 2102Ep cells. Previous evidence demonstrates the induction of EMT and the acquisition of neural crest cell traits in hESCs after SOX2 knock down and the central roles of SOX2 in this type of cell fate determination have been previously suggested [22, 50]. This phenotypic change is particularly evident in 2102Ep cells, which produced more altered markers than NTera-2 cells. In cancer, EMT is a process of transdifferentiation by which epithelial cells lose their cell-cell adhesion and adopt a migratory mesenchymal phenotype enabling them to metastasise to other tissues. Unexpectedly, some established EMT inhibitors were upregulated in NTera-2 (CDH1/E-cadherin) and 2102Ep cells (KRT19 and HAS2) [51, 52]. This observation will be further touched upon in the Discussion section. Among the 26 EMT markers, SNAI2/Slug and CDH2/N-cadherin have been previously shown to be transcriptionally activated by SOX2 during Type 3 and Type 1 EMT respectively. In our data, these genes are upregulated after SOX2 knock-down, suggesting an opposite transcriptional relation to SOX2 [10, 53]. On the other hand, T/Brachyury and EOMES were previously found to be repressed by SOX2 in Type 1 EMT, which is in concordance with our findings [54, 55].

We performed Monte Carlo analysis on the 2102Ep and NTera-2 differential gene expression datasets and cross-referencing with the results with the differential miRNA expression results revealed 10 miRNAs in 2102Ep cells *(mir-26a, miR-28, miR-30c, miR-148a, miR-200b, miR-517b, miR-518a-3p, miR-518b, miR-518c, miR-518f)* and two miRNAs in NTera-2 cells *(miR-200c and miR-367)* to be potential master regulators of their inversely regulated target genes. Certain human miRNA families are broadly conserved across many vertebrate species, while the evolutionary conservation of others is limited to mammals or mammalian species of close common ancestry. The corollary is that poorly conserved miRNAs may bind to poorly conserved target sites. To maximise the probability of identifying true miRNA targets we limited our scope to target sites of equal conservation to their respective miRNAs. While miR-26a, miR-30c, miR-148a, miR-200b, miR-200c and miR-367 are broadly conserved across vertebrate species, miR-28 is conserved only in mammals and miR-517b, miR-518f, miR-518b, miR-518c, miR-518a-3p, all as members of the C19MC polycistron, are found only in primates.

To identify high-probability gene targets we further filtered the results by microT-CDS and miRanda (August 2010 release) cross-analysis and removed any targets that were not predicted by either of these tools (2 cases) [56, 57]. The results are found in Table 2 and with the added prediction scores in Additional file 5: Table S5. The results reveal a combined set of 128 miRNA-target interactions with 85 unique genes potentially regulated by our significant set of miRNAs. Of these, 99 miRNA-target interactions are predicted by all three target prediction tools used in this study governing a high-confidence set of 75 unique genes, 19 of which have two or more high-confidence miRNA interactions. This portrays a dense network of interlinking miRNA-target regulation containing many previously validated miRNA targets (expressed in bold in Table 2) and many potentially new targets.

**Table 2:** **Significantly represented miRNAs in 2102Ep and NTera-2 data with their associated targets**

Cell line	miRNA	Fold change	Targets
2102Ep	miR-148a	-3.63	**ARRDC3** [58], ATP2B4, CHD7, ELMO1, FAM123B, H2AFY, HDGF, *HMGA2*, IL6ST, **ITGA5** [59], ***ITGB8*** [59], LIX1, NRP1, PREX1, *TEAD1*, TMEM54
	mir-26a	-1.73	ADAM19, AMOT, CDH2, CDH11, CSNK1G1, ENC1, ENPEP, HAS2, **HMGA2** [60], **HOXA5** [61], HOXA9, *LHX1*, ITGA5, ITGB8, **LEF1** [62], *LIFR*, **MAP2** [63], NID1, NRIP1, PLXNA2, *PRTG*, SSFA2, TFAP2A, USP3, WNT5A, ZSWIM6
	miR-200b	-1.48	*AHNAK*, ARRDC3, CDH11, CNKSR3, CNTFR, **EFNA1** [64], *EMP1*, **FN1** [65], **GATA2** [66], GLI3, HEG1, HOXA5, KIAA0101, MAP2, *MBNL3*, NKD1, NRIP1, NRP2, NTF3, PLXNA2, PRTG, ST6GALNAC5, *TEAD1*, **TFAP2A** [67], WWC3, **ZEB2** [68]
	miR-30c	-1.23	ACTC1, ADAM19, ADAMTS9, AHNAK, CAMK2N1, *CHD7*, CSNK1G1, CYP24A1, ELMO1, FAM123B, GRHL2, ITGA5, KIAA1024, *LHX1*, LIFR, MBNL3, NID1, NRIP1, *NRP2*, PLXNA2, *PRTG*, RARG, RASGRP3, RHOB, **SNAI1** [69], SNAI2, TBL1X, *TEAD1*, TIMP3, ZEB2, ZSWIM6
	miR-28	1.71	*DPF1* , IQSEC2, PRUNE2
	miR-517b	-1.94	*CACNG4*, *HOXA5*, NKD1, *PRTG*, *ZEB2*, *ZNF436*
	miR-518a-3p	-2.47	*FRAS1*, *IL13RA1*, *TEAD1*, *TGFBR3*
	miR-518b	-2.06	
	miR-518c	-3.33	
	miR-518f	-1.87	
NTera-2	miR-200c	-1.80	AHNAK, CITED2, *MBNL3*, **NTF3** [70], PLXNA2, PRTG, **SEMA6D** [71], YPEL2
	miR-367	-1.46	ATP2B4, CADM2, EOMES, HAND1, *MBNL3*, *PKDCC*, *SEMA6D*, SESN3

Genes in bold are previously confirmed targets of their respective miRNAs which also showed up in our data. Genes in cursive style are predicted by TargetScan and either microT-CDS or miRanda (August 2010 Release). All other genes were predicted by all three prediction tools.

Many genes related to EMT pathways were found to be regulated by several differentially expressed miRNAs. Of these, members of the miR-200 family have been the most extensively studied in this context [72]. We discovered that a group of 11 miRNAs in Table 2, all of which were downregulated, could target 14 differentially expressed EMT-related genes from both cell lines combined. Four of these miRNAs, miR-200b, miR-200, miR-30c and miR-148a, are established inhibitors of EMT and metastasis by targeting ZEB1 and ZEB2 (miR-200b/200c), TWF1 and VIM (miR-30c) and mesenchymal-to-epithelial transition (MET) (miR-148a) [68, 73, 74]. Additionally, miR-26a has targets that were verified in non-EMT studies, but which have independently established functions in EMT and metastasis. These include HMGA2 and LEF1 (miR-26a) [60, 62, 75]. Figure 3C illustrates a network of validated and novel SOX2-miRNA-target interactions found in this study. Some of these interactions are further detailed in the Discussion section.

### Meta-analysis of miRNAs with SOX2 binding sites in their promoter regions

To provide further insight into the miRNA transcriptome under direct control by SOX2, we cross-analysed our profiling data with existing genome-wide SOX2 ChIP data.

We first performed a meta-analysis of SOX2 transcription factor binding sites (TFBSs) in proximity of miRNA transcription start sites (TSSs). At the time of this study, no previously published SOX2 ChIP experiments were performed for hECC lines. Because of this we adopted a similar strategy used by Greber *et al*. and performed analysis with SOX2 ChIP data from hESCs as a substitute [23]. The assumption that SOX2 binds similar genomic loci in both hECCs and hESCs was based on the evidence supporting a phenotypic similarity between these cell types and further supported by functional studies which show that SOX2 has highly comparable roles in the maintenance and differentiation of these cell lines [23, 76]. Some differences do exist between hECC and hESCs on the genetic and transcriptomic level, but current research accepts that this is limited to the expression and alternative splicing of only a relatively small number of genes [77].

Two whole-genome hESC SOX2 ChIP datasets were included in the analysis; those previously published by Boyer *et al*., and Lister *et al.* [11, 12] *.* Lister *et al.* performed ChIP-Seq of the whole genome, while Boyer *et al.* used a more limited ChIP-on-chip based method which covered the -8 kb to +2 kb genomic regions relative to transcription start sites of 17,917 annotated genes.

Secondly, we compiled a list of TSSs from two resources: a dataset of miRNA TSSs identified by Bulik-Sullivan *et al*. and all host gene and miRNA TSSs from the miRStart database [78, 79]. All miRNAs with SOX2 binding sites 5 kb upstream or downstream from their miRNA TSS and, where applicable, their host gene TSS, were compiled and included in further analysis.

In total, 71 unique miRNA precursors were found to have one or more SOX2 binding sites (see Additional file 6: Table S6). We compared this set of miRNAs with all miRNAs that we found to be significantly down or upregulated in the 2102Ep and NTera-2 cell lines. From this analysis, 11 deregulated mature miRNAs in 2102Ep cells and 12 in NTera-2 cells were found to have proximal SOX2 binding sites as shown in Table 3. In NTera-2 cells, all these miRNAs were downregulated, while in 2102Ep cells this transcriptional activity was mixed. Only miR-135b* and miR-181a-2* were commonly listed for both 2102Ep and NTera-2 cells. This signifies a difference between the two hECC lines in response to SOX2 downregulation.

**Table 3:** **MiRNAs with SOX2-binding sites in hESCs which were deregulated in 2102Ep and NTera-2 cells**

	Precursor	Mature miRNA	Fold-change	Distance to TSS	Expression EMT/MET	Validated targets × hECC data
**2102Ep**	hsa-mir-9-2	miR-9*	5.16	1887		
	hsa-mir-9-2	miR-9	3.83	1887	Up in EMT [80]	CDX2, ID2
	hsa-mir-181a-2	miR-181a-2*	3.05	-467		
	hsa-mir-424	miR-424	2.87	3042	Up in EMT [81]	
	hsa-mir-301a	miR-301	1.78	55		
	hsa-mir-182	miR-182	-2.08	-4286	Up in MET [82]	RARG, SNAI2
	hsa-mir-942	miR-942	-2.10	-126		
	hsa-mir-183	miR-183*	-2.32	-4286		
	hsa-mir-183	miR-183	-2.90	-4286	Down in EMT [83]	SNAI2
	hsa-mir-135b	miR-135b*	-3.04	4448		
	hsa-mir-766	miR-766	-5.04	-1234		
**NTera-2**	hsa-mir-19b-2	miR-19b	-1.35	-932		PRUNE2
	hsa-mir-22	miR-22	-1.58	-186	Up in EMT [84]	
	hsa-mir-324	miR-324-5p	-1.91	-2058		AHNAK
	hsa-mir-124-2	miR-124	-1.98	-5	Down in EMT [85]	
	hsa-mir-22	miR-22*	-2.02	-186		
	hsa-mir-7-1/7-3	miR-7	-2.27	-2262	Down in EMT [86]	
	hsa-mir-135b	miR-135b*	-2.33	4448		
	hsa-mir-135b	miR-135b	-2.56	4448		
	hsa-mir-181a-2	miR-181a	-2.61	-467	Up in EMT [87]	NANOG
	hsa-mir-577	miR-577	-3.31	1455		
	hsa-mir-181a-2	miR-181a-2*	-4.47	-467		
	hsa-mir-1251	miR-1251	-7.90	1717		

None of the miRNAs listed in Table 3 have been validated before as transcriptional targets of SOX2, revealing a novel network of miRNAs potentially directly regulated by SOX2 in pluripotent cells. In general, few miRNAs have so far been identified as direct transcriptional targets of SOX2 in human cells. Previous research efforts have focused on its regulation of the ESC-specific miR-302-367 cluster and recently SOX2 has been implicated in a direct negative feedback loop with the miR-200 family [88–90]. Despite the significant deregulation of miR-302c and miR-200b in 2102Ep cells and miR-200c and miR-367 in NTera-2 cells, none of these miRNAs were identified as SOX2 transcriptional targets in our data. The false negative result for the miR-302-367 cluster can be explained by the absence of identifiable TSSs for this cluster. However, two SOX2 binding sites were found in a <2000 bp proximity of the miRNA precursors, confirming previous reports. No SOX2 binding sites were in proximity to the TSSs of miR-200b and miR-200c as mapped by Boyer *et al*. and Lister *et al*.

A literature search revealed a substantial overlap of miRNAs potentially controlled by SOX2 as listed in Table 3 and miRNAs functionally linked to EMT or its counterpart, MET, in cancer or embryonic stem cell lines. Table 3 column ‘Expression during EMT/MET’ indicates the direction of differential expression during EMT or MET. With the exception of miR-22 and miR-181a in NTera-2 cells, the differential expression of all miRNAs listed in Table 3 correspond with their reported expression during EMT or MET.

Additionally, with the aid of miRTarBase, a repository of validated miRNA-target interactions, genes deregulated in 2102Ep and NTera-2 cells after SOX2 knock-down were included as previously validated targets [91]. Only interactions validated with strong evidence (reporter assay, Western blot or qPCR) were included. Notably, SNAI2/Slug, an important EMT gene, is upregulated in 2102Ep cells, while its previously validated miRNAs, miR-182 and miR-183, are downregulated. Both miRNA are members of the same miR-183-96-182 cluster in the genome. This potentially presents alternative route through which SOX2 controls SNAI2/Slug transcription (Figure 3C).

## Discussion

SOX2 is a major pluripotency marker and oncogene. Although previous studies have addressed the SOX2 transcriptional profile of protein-coding genes in both hESCs and hECCs [11, 12, 23], no study has yet profiled the SOX2 miRNA profile in human pluripotent cells. Successful previous attempts have mapped the direct transcriptional miRNA network of SOX2 in mouse ESCs and human GBM, but no integrated differential gene and miRNA expression analysis was performed and none exist in human pluripotent stem cells [1, 26]. The basis of this study was to attempt such analysis and to provide a platform for future SOX2 and pluripotency-linked miRNA-target discovery and validation.

ECCs have been previously used as a model to study cancer stemness, ESCs and embryonic development [37, 38, 40, 42, 77, 92]. As CSCs of embryonal carcinoma and teratocarcinoma, they have been subjects of cancer stemness studies, while their phenotypical similarities with ESCs have kept ECCs interesting to researchers who study embryonic development and ESC biology. Recently, these fields have converged in research which utilises ECCs as a model to investigate ESC and iPSC tumourgenicity and cancer cells expressing embryonic biomarkers [42, 77, 93].

### MiRNA profiling after SOX2 knock-down reveals a distinct phenotypes in 2102Ep and NTera-2 cells

We profiled the differential expression of 754 miRNAs in two SOX2 knock-down ECC lines. To our best knowledge, this is the first study to map a SOX2-linked miRNA network by a high-throughput method in human pluripotent cell lines. MiRNA profiling revealed the significant deregulation 99 and 62 unique mature miRNAs in 2102Ep and NTera-2 cells, of which only 18 were common between these groups, with four miRNAs of this subset oppositely deregulated. The large difference in miRNA response to SOX2 knock-down signifies the phenotypic differences between the 2102Ep and NTera-2 cell lines which is further supported by the different miRNA profiles between undifferentiated 2102EP and NTera-2 cells.

However, the miRNA profiles in both cell lines showed certain features expected in hESCs after SOX2 silencing. In 2102Ep cells, several ESC-specific miRNAs were downregulated, most notably 28 members of the C19MC polycistron. In NTera-2 cells, this cluster is expressed, but only two of its miRNAs (miR-517c and miR-522) were downregulated. C19MC is located on chromosome 19q13.42 and conserved to primates only. In normal tissue, its miRNAs are specifically expressed in ESCs, ECCs and placental tissue. Although the precise function of C19MC is still quite elusive, its expression is rapidly lost during early ESC differentiation [94]. However, C19MC re-expresses in several cancer types and in particular embryonal brain tumours in which it activates an early developmental program by driving global methylation changes [95]. The downregulation of C19MC after SOX2 knock-down in 2102Ep cells and its its apparent resistance to this in NTera-2 cells aligns the phenotype of the former cell line closer to that of hESC differentiation than the latter.

### MiRNA profiling after SOX2 knock-down expands the autoregulatory network of core pluripotency factors

The differential expression of three miRNAs, miR-9*, miR-145 and miR-126, which have previously been validated to target SOX2, suggests the possible existence of novel autoregulatory loops between SOX2 and miRNAs it directly or indirectly regulates. MiR-9*, which is upregulated in 2102Ep (Figure 2A), and miR-145 and miR-126, which are upregulated and downregulated in NTera-2 respectively (Figure 2B), all have been validated to repress SOX2 [18, 96–98]. While the downregulation of miR-126 points towards a negative feedback mechanism, the upregulation of miR-9*, which has a proximal SOX2 binding site in its promoter region (Table 3), and miR-145 indicates the existence of positive feedback loops. In fact, Fang *et al*., who found similar deregulation of miR-145 upon the knock down of SOX2 in GBM cells, proposed this association [1].

Expanding these autoregulatory networks beyond the direct targeting of SOX2 reveals additional regulatory connections between SOX2-targeted miRNAs (Table 3) and regulators of SOX2 expression. The pluripotency factor NANOG reciprocally induces SOX2 expression and both can cooperatively enhance their own expression in stem cells, including ESCs, ECCs and CSCs [99, 100]. As expected, NANOG was similarly downregulated in both ECC lines in this study. In hematopoietic stem cells, miR-181a-2*, a member of the miR-181 family and deregulated in both 2102Ep and NTera-2 cells, can directly target NANOG [101]. The expression of the miR-181 family increases during early hESC differentiation and has previously been identified as regulators of stem cell differentiation, including that of hESCs and CSCs [102–105]. We found that the miR-181a/-2a* precursor contains a SOX2 binding site in close proximity to its TSS (Table 3), suggesting its direct regulation of these miRNAs. MiR-181a-2* was upregulated in 2102Ep cells, while its complement strand miR-181a was similarly upregulated just outside the minimal threshold of statistical significance (not shown). If miR-181a-2* targets NANOG during 2102Ep differentiation, this would present an alternative mechanism of NANOG regulation by SOX2 in this ECC line, and possibly other cell lines.

### Integrated analysis reveals a distinct EMT miRNA-target regulatory network

From a statistical cross-analysis of miRNA and gene profiling data we identified 12 miRNAs that controlled a combined set of 85 deregulated genes, of which 75 were predicted by three computational target prediction tools. This set was enriched with a subgroup of 17 genes involved in EMT (Figure 3C). SOX2 has recently been directly linked to EMT and metastasis in cancer, and as an oncogene is considered to be a promoter of EMT during disease progression [3, 106–108]. Although the precise mechanisms by which SOX2 contributes are still quite elusive, its cancer stem cell regulatory and EMT functions probably overlap. So far, one study found that its transcriptional regulation of the Wnt/β-catenin pathway was a contributing factor [108]. Recent evidence suggests that EMT could attribute to the generation of CSCs, possibly further implicating SOX2 in a network of tumorigenesis and progression though its expression in a cancer cell subpopulation [109, 110].

From our results we present an expanded network of miRNA interactions, directly and indirectly regulated by SOX2, that could govern EMT during embryonic development and in CSCs. The significant representation of known and putative miRNA inhibitors of EMT with validated EMT targets (miR-200b, miR-200c, miR-30c, miR-148a and miR-26a) provides functional significance to the wider SOX2-regulated miRNA-target network revealed in this study. Furthermore, independent from the statistical target analysis, miR-9/9*, a highly characterised promoter of EMT and upregulated in 2102Ep cells, has a SOX2-binding site in its promoter region (Table 3), further revealing a SOX2-linked miRNA EMT network [80].

This network includes highly characterised mesenchymal markers such as ZEB2, LEF1, FN1 (fibronectin), CDH2 (N-cadherin), SNAI1 (Snail) and SNAI2 (Slug). Nearly all genes in this upregulated group of genes are promoters or effectors of EMT. The exception is CDH1/E-cadherin in NTera-2 cells, which normally downregulates during EMT, while it is upregulated in our dataset. The latter cannot be fully explained without further experimentation. Overall, the results demonstrate a miRNA link between SOX2 and EMT-related genes in 2102Ep cells. In a recent study, Cimadamore *et al*. demonstrate that the differentiation of hESCs to sensory neurons rely on a transient up- and downregulation of SOX2 expression [22]. SOX2 expression reduces during the initial differentiation and EMT of hESCs to neural crest cells after which SOX2 is re-expressed during the differentiation of these cells towards neuronal progenitors. The induction of EMT genes in 2102Ep cells represent the first phase of this differentiation program and the detailed network of miRNA-target interactions we present in our study could assist this process. Furthermore, despite their pluripotency, NTera-2 cells appear have a limited capacity of undergoing this transformation or perhaps represent a more progressed phenotype along this differentiation program as suggested by the upregulation of CDH1/E-cadherin and the downregulation of NRP1. The induction of a combined gene and miRNA EMT programme by SOX2 knock-down in 2102Ep cells suggest the activation of a form of Type 1 EMT.

### MiRNA promoter analysis links SOX2 transcriptional function to validated EMT miRNAs

The characterisation of a miRNAs with SOX2 binding sites in their promoter region yielded a subset of deregulated miRNAs in 2102Ep and NTera-2 that are highly probable to be transcriptionally regulated by SOX2. 9 miRNAs have been previously validated as functional activators or inhibitors of EMT or MET. In 2102Ep cells, inhibitors of EMT, miR-9 and miR-424, and an activator of MET, miR-182, are all upregulated. Conversely, an inhibitor of EMT, miR-183, is downregulated. This further supports the hypothesis of SOX2 as a regulator and an inhibitor of Type 1 EMT in 2102Ep cells. In NTera-2 cells, miR-124-3p, miR-7, miR-22 and miR-181a are downregulated. However, only miR-124-3p and miR-7 are inhibitors of EMT, while miR-22 and miR-181a are activators. As indicated by the differential expression of certain genes in NTera-2 cells, this could be representative of a more progressed phenotype. This has been suggested before, for despite their pluripotent nature, NTera-2 cells appear to be intrinsically primed towards neural differentiation, a process also governed by SOX2 [22, 111, 112]. Unbiased screening of the miRNAs in Table 3 with validated targets from miRTarBase yielded only few genes that were deregulated in 2102Ep and NTera-2 cells. It is possible that functional response of targets of these miRNAs is delayed or that these miRNAs confer their functionality through translational repression. Furthermore, curated databases such as miRTarBase only represent a fraction of the results in the literature and, as such, several validated targets may been missed.

A direct association between master regulator miRNAs (Table 2) and miRNAs with SOX2 binding sites in their promoter regions (Table 3) could not be found. This may be due to the high stringency of statistical approach to identifying master regulator miRNAs taken in this study rather than an alternative SOX2 binding profile to miRNA promoter regions. Despite this, the master regulators, miR-200b, miR-200c and miR-367, have been previously established transcriptional targets of SOX2, indicating that this transcription factor could have a large influence on the targets of these miRNAs through their genomic regulation [88–90].

## Conclusion

In this study we profiled the SOX2-linked miRNAome and its associated regulatory network in pluripotent and nullipotent cancer stem cells. The findings add to a growing body of results that map the cell and context specific multifunctionality of SOX2 in the maintenance and direction of stem cell phenotypes. The results presented in this study suggest a miRNA link between SOX2 and Type 1 EMT markers in the 2102Ep ECC line. The apparent role of SOX2 as an inhibitor of EMT during embryonic development is opposite of its role as a promoter EMT in metastatic neoplasms, even though many of the same miRNAs are involved. Future research could further and individually validate the presented miRNAs and targets in relation to SOX2-linked EMT, embryonic development and cancer.

## Methods

### Cell culture and functional transfections

For this study we compared two hECC lines; 2102Ep and NTera-2 (kind gifts from Prof. Peter Andrews, University of Sheffield). These cell lines have a high turn-over and remain in an undifferentiated state at high density. The cells were grown in growth medium containing high-glucose DMEM supplemented with 10% FCS and 2% penicillin-streptomycin (Lonza, Switzerland). For experimentation, six-well plates were seeded 24 hours prior to oligonucleotide transfection in growth medium without antibiotics. This study did not involve human samples or data and as such did not require ethical approval.

SOX2 knock-down was performed with a pre-designed Silencer^®^ Select siRNA *(s13295, Life Technologies, USA)* with a sense sequence of AGUGGAAACUUUUGUCGGATT and an anti-sense sequence of UCCGACAAAAGUUUCCACUCG. In NTera-2 cells, functional transfections were performed as per manufacturers’ protocols with 30 nM siRNA and Lipofectamine^®^ RNAiMAX^®^*(Life Technologies, USA)*. For 2102Ep cells, 15 nM siRNA with Lipofectamine ^®^ 2000 *(Life Technologies, USA)* was sufficient. As a negative control, samples transfected with the non-targeting siRNA, Silencer^®^ Select Negative Control #1 were included. Vehicle controls, absent of any siRNA, and non-transfected controls, absent of any transfection agent or siRNA, were also included to test the effects of the transfection components. The cells were forward transfected in a serum-free medium containing Opti-MEM^®^ I, transfection agent and siRNA for 6 hours at 37°C. The medium was replaced with normal growth medium and the cells were incubated in appropriate conditions for 72 hours with a daily medium refresh after which the cells were harvested for Western blot and RNA expression analysis.

### Validation of SOX2 knock-down

Successful knock down of SOX2 by siRNA was assessed by measuring relative mRNA and protein levels with qRT-PCR and Western blot respectively. Briefly, total RNA and protein samples were isolated and purified from the cells using the miRVANA^®^ PARIS system (Ambion, USA). MRNA expression was determined using the the TaqMan^®^ qRT-PCR system (Applied Biosystems, USA) as per manufacturer’s protocols. Relative quantification with the 2^-ΔΔCt^ method, as summarised by Livak and Schmittgen [113], was used to compare mRNA expression in the functional samples compared to the negative controls. Primer/probe pairs were used to measure the expression of SOX2 *(Hs00602736_s1, Applied Biosystems, USA)* and an endogenous normalisation control, GAPDH *(4331182, Applied Biosystems, USA)*. The RNA samples were used for down-stream gene and miRNA analysis. Western blot was performed with standard methods, using the enhanced chemiluminescence developed previously by Haan and Behrmann [114] *.* For probing, primary antibodies were used against SOX2 *(ab75485, AbCam, UK)* and the endogenous normalisation control, GAPDH *(ab8245, AbCam, UK)*.

### MicroRNA expression analysis

Whole-miRNAome analsysis was performed with the TaqMan^®^ Array Human microRNA system *(Applied Biosystems, USA)* as per manufacturer’s protocols. This provided a high-throughput method to quantitatively screen the expression of 754 unique miRNAs, covering the broad majority of human miRNAs in the Sanger miRBase v14 miRNA database [44]. For this, cDNA prepared from the sample RNA underwent qRT-PCR on a pair of 384-well plates containing lyophilised primer/probe pairs. Three biological replicates were analysed for both functional and negative control samples. To enhance the detection of low-concentration miRNAs, a pre-amplification step was included using the TaqMan PreAmp Master Mix with the Megaplex PreAmp Primers *(Applied Biosystems, USA)*. The manufacturer’s DataAssist 3.0 software was used to batch-analyse the expression results. MiRNAs that were deregulated with a P-value of ≤0.05 were included in the final dataset.

### Gene array analysis

The Affymetrix GeneChip Human Gene 1.0 ST array platform was used to study the differential expression of 28,132 transcripts. RNA integrity was measured with the 2100 Bioanalyzer microfluidics station *(Agilent, USA)* according to manufacturer’s protocols. The preparation and labelling of sense cDNA was performed to manufacturers’ protocols with the WT Expression Kit *(Ambion, USA)* and the GeneChip WT Terminal Labeling kit *(Affymetrix, USA)*. Hybridisation, washing and staining was performed with Hybridization, Wash and Stain Kit *(Affymetrix, USA)*. Scanning and quality control analysis was performed on the GeneChip Scanner 3000 7G *(Affymetrix, USA)* with the manufacturer’s propriety software using the robust multi-array average analysis. Three biological replicates were performed with both the functional and negative control samples. Computational expression analysis was performed using the open-source R *(CRAN)* package, Bioconductor 2.9 with the limma (Linear Models for Microarray Data) add-on [115]. Gene array results were subsequently validated with qRT-PCR. To reduce the chance of false positive results from downstream analysis, differentially expressed genes included in the final dataset were those that were ≥2-fold deregulated with a P-value of ≤0.01 and false discovery rate value of ≤0.05.

### Gene and miRNA annotation analysis and network mapping

To categorise genes by function, we primarily performed gene ontology analysis using DAVID Bioinformatics Resources 6.7 [49]. On significant findings from DAVID and on unannotated genes we performed further literature searches for gene functionality using a defined list of MeSH terms which included those related to ESCs, EC cells, CSCs, differentiation, embryonic development, EMT and cancer. Figure 3C was compiled from miRNA-target interactions and SOX2 binding sites predicted in this study. Figure 3C was further enriched with previously validated transcriptional and post-transcriptional interactions between miRNAs, EMT genes and SOX2 as described in Table 2, Table 3 and the text.

### Integrated gene and miRNA cross-analysis

To find a functionally enriched set of miRNAs that are predicted to target the set of significantly deregulated genes, we adapted a Monte Carlow algorithm previously successfully used by Stone *et al.* and Bulik-Sullivan *et al.* [47, 79] *.* Briefly, genes with at least one miRNA target site in TargetScan 6.0 were randomly selected from the human genome. The number of random genes selected was equal to the size of each group of upregulated or downregulated genes in 2102Ep and NTera-2 cells. From this set the number of genes that each human miRNA in the TargetScan 6.0 database was predicted to target was calculated. From this a weighted score was calculated based on the number of predicted target site per 3′UTR, the distance between target sites and the type of predicted hybridisation. This simulation was repeated 10,000 times to calculate a background distribution of the number of genes each human miRNA is predicted to target. This distribution was subsequently used to calculate an empirical p-value for the predicted number of upregulated or downregulated target genes in 2102Ep and NTera-2 cells. To account for the differences in average 3′UTR length between these four groups and their respective groups of randomly selected genes, the number of predicted target genes was normalised against the average 3′UTR length of the randomly selected genes. To establish additional confidence in the probability of the generated miRNA-target interactions, all miRNAs were further screened for their targets using the microT-CDS and miRanda (August 2010 release) computational target prediction tools [56, 57]. For microT-CDS a threshold of ≥0.4 was set (a ‘medium-good’ setting according to the authors), while for miRanda a mirSVR maximum of ≥0.1 (a ‘good’ setting according to the authors) was applied.

### Supporting data

The data sets supporting the results of this article are included within the article and its Supplementary Tables.

## Electronic supplementary material

Additional file 1: Table S1: Differentially regulated miRNAs in 2102Ep and NTera-2 cells. All differentially regulated miRNAs after SOX2 knock-down in 2102Ep and NTera-2 cells. (XLSX 17 KB)

Additional file 2: Table S2: MiRNA expression in undifferentiated NTera-2 cells compared to undifferentiated 2102Ep cells. All differentially regulated miRNAs between the two hECC cell lines in their undifferentiated, unaltered state. (XLSX 18 KB)

Additional file 3: Table S3: Genes deregulated in 2102Ep and NTera-2 cells after SOX2 knock-down. All genes differentially regulated in 2102Ep and NTera-2 cells three days after SOX2 knock-down. Including a table with genes deregulated in both cell lines. (XLSX 123 KB)

Additional file 4: Table S4: DAVID Bioinformatics Resources 6.7 Output for 2102Ep and 2102Ep cells. Gene annotation analysis of all deregulated genes in 2102Ep and NTera-2 cells by DAVID Bioinformatics Resources 6.7. (XLSX 46 KB)

Additional file 5: Table S5: Predicted miRNA-target interactions by master regulator miRNAs. Master regulator miRNAs and their targets as predicted by our statistical analysis and further enhanced by microT-CDS and miRanda prediction with prediction scores included. (XLSX 15 KB)

Additional file 6: Table S6: MiRNAs with proximal SOX2 TFBSs. All miRNAs with TSSs close to SOX2 TFBSs as defined by the criteria outlined in the study. The table includes miRNA TSS coordinates and SOX2 TFBS coordinates. (XLSX 12 KB)

## Abbreviations

ChIP: Chromatin immunoprecipitation
ChIP-seq: ChIP-sequencing
CSC: Cancer stem cell
ECC: Embryonal carcinoma cell
EMT: Epithelial-to-mesenchymal transition
ESC: Embryonic stem cell
GBM: Glioblastoma multiforme
hECC: Human embryonal carcinoma cell
hESC: Human embryonic stem cell
iPSC: Induced pluripotent stem cells
MET: Mesenchymal-to-epithelial transition
NC: Non-targeting siRNA
NTC: Non-transfected control
oncomiR: Oncogenic miRNA
qRT-PCR: Quantitative real-time PCR
siSOX2: SOX2 siRNA
SOX: SRY-related HMG-box
TFBS: Transcription factor binding site
TSS: Transcription start site
VC: Vehicle control.
